# Scaffold fragmentation and substructure hopping reveal potential, robustness, and limits of computer-aided pattern analysis (C@PA)

**DOI:** 10.1016/j.csbj.2021.05.018

**Published:** 2021-05-10

**Authors:** Vigneshwaran Namasivayam, Katja Silbermann, Jens Pahnke, Michael Wiese, Sven Marcel Stefan

**Affiliations:** a Department of Pharmaceutical and Cellbiological Chemistry, Pharmaceutical Institute, University of Bonn, An der Immenburg 4, 53121 Bonn, Germany; b Department of Neuro-/Pathology, University of Oslo and Oslo University Hospital, Sognsvannsveien 20, 0372 Oslo, Norway; c LIED, University of Lübeck, Ratzenburger Allee 160, 23538 Lübeck, Germany; d Department of Pharmacology, Faculty of Medicine, University of Latvia, Jelgavas iela 1, 1004 Rīga, Latvia; e Department of Bioorganic Chemistry, Leibniz-Institute of Plant Biochemistry, Weinberg 3, 06120 Halle, Germany; f Cancer Drug Resistance and Stem Cell Program, University of Sydney, Kolling Builging, 10 Westbourne Street, Sydney, New South Wales 2065, Australia

**Keywords:** ABC transporter, ATP-binding cassette transporter, ATP, adenosine-triphosphate, BCRP, breast cancer resistance protein (ABCG2), calcein AM, calcein acetoxymethyl, C@PA, computer-aided pattern analysis, F1–5, pharmacophore features 1–5, IC_50_, half-maximal inhibition concentration, MDR, multidrug resistance, MRP1, multidrug resistance-associated protein 1 (ABCC1), MOE, molecular operating environment, P-gp, P-glycoprotein (ABCB1), SEM, standard error of the mean, SMILES, simplified molecular input line entry specification, Tc, Tanimotto coefficient, Well-studied ABC transporters, ABCB1 (P-gp), ABCC1 (MRP1), ABCG2 (BCRP), Under-studied ABC transporters (*e.g.*, ABCA7), Triple / multitarget / broad-spectrum / promiscuous inhibitor / antagonist, Pan-ABC inhibition / antagonism / blockage (PANABC), Pattern analysis (C@PA), Multitarget fingerprints, Alzheimer's disease (AD), Multidrug resistance (MDR)

## Abstract

Computer-aided pattern analysis (C@PA) was recently presented as a powerful tool to predict multitarget ABC transporter inhibitors. The backbone of this computational methodology was the statistical analysis of frequently occurring molecular features amongst a fixed set of reported small-molecules that had been evaluated toward ABCB1, ABCC1, and ABCG2. As a result, negative and positive patterns were elucidated, and secondary positive substructures could be suggested that complemented the multitarget fingerprints. Elevating C@PA to a non-statistical and exploratory level, the concluded secondary positive patterns were extended with potential positive substructures to improve C@PA’s prediction capabilities and to explore its robustness. A small-set compound library of known ABCC1 inhibitors with a known hit rate for triple ABCB1, ABCC1, and ABCG2 inhibition was taken to virtually screen for the extended positive patterns. In total, 846 potential broad-spectrum ABCB1, ABCC1, and ABCG2 inhibitors resulted, from which 10 have been purchased and biologically evaluated. Our approach revealed 4 novel multitarget ABCB1, ABCC1, and ABCG2 inhibitors with a biological hit rate of 40%, but with a slightly lower inhibitory power than derived from the original C@PA. This is the very first report about discovering novel broad-spectrum inhibitors against the most prominent ABC transporters by improving C@PA.

## Highlights

• Exploratory changes in substructure patterns are well tolerated by C@PA.
• Extended positive substructures support prediction capability.
• Increased biological hit rate of 40% for multitarget pan-ABC transporter inhibition.
• Contribution to major understanding of pattern analysis and multitarget activity.
• Pan-ABC transporter inhibitors as tool for elucidation of multitarget binding site.

## Graphical abstract

**Figure:**
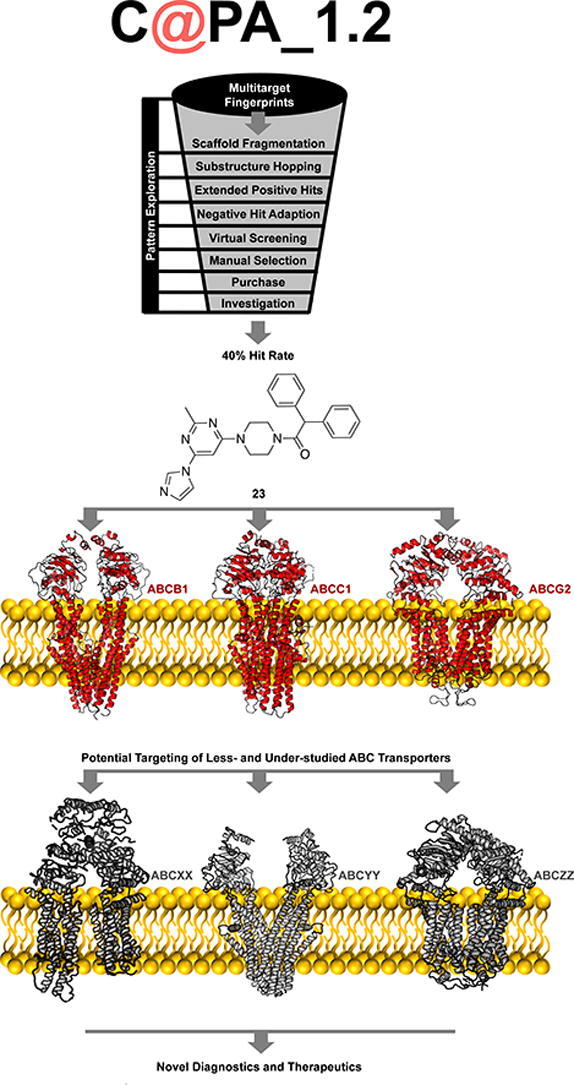

## 1 Introduction

ATP-binding cassette (ABC) transport proteins are ubiquitously present in the human body [1], [2], [3], [4], and hence, promote solute and drug distribution, influencing their pharmacokinetic. However, dysfunction of these efflux pumps contribute also to major human diseases. Amongst these diseases are neurological disorders [2], such as Alzheimer’s disease [2], [5], [6], [7], [8], metabolic diseases and related illnesses [9], such as atherosclerosis [9], but also malignant diseases, such as multidrug-resistant cancer [3], [10], [11], [12], [13], [14]. For example, half of the A and G subclasses of ABC transporters have been identified as contributors in Alzheimer’s disease, correlating their downregulation or defective function with a negative disease development [6], [7]. Another example is multidrug-resistant cancer, where the vast majority of ABC transporters has been associated with the multidrug resistance (MDR) phenotype [12], [13], and many transporters were indeed found to export applied antineoplastic agents out of cancer cells, ultimately protecting these from cell death [11], [14].

Unfortunately, only a small fraction of the 49 existing ABC transporters can be considered as well-studied, in particular ABCB1 [8], [14], [15], [16], [17], [18], [19], ABCB11 [20], [21], [22], ABCC1 [1], [4], [14], [15], [17], [23], [24], and ABCG2 [14], [15], [17], [25]. Less-studied ABC transporters that have found much less attention are ABCC2, ABCC4–5, and ABCC10 [1], [4], [14], [24], [26], as well as – to a lesser extend – ABCA1 [27], [28], [29], [30], ABCB4 [14], ABCC3 [1], [4], [14], [24], as well as ABCC7–9 and ABCC11 [1], [4], [31], [32]. The remaining 34 transporters can be considered as under-studied which cannot be addressed by small-molecule modulators besides very rare exceptions. However, small-molecules would represent a potential tool to monitor, influence, and study these transporters for (i) a general understanding of their mechanism of action, and more importantly, for (ii) their exploration as potential pharmacological targets to develop innovative diagnostics and therapeutics.

As a logical consequence, the number of synthetic approaches to gain novel lead structures and potent modulators of ABC transporters is also very unequally distributed amongst the ABC transport proteins, and very scarce for under-studied ABC transporters. While many hundreds of small-molecule modulators of ABCB1 [14], [15], [16], [17], [18], [19], ABCC1 [1], [4], [14], [15], [17], [23], [24], and ABCG2 [14], [15], [17], [25] exist, synthetic approaches to target other ABC transporters have barely been reported. Rare exceptions are, for example, ABCC4 [33], [34], ABCC8 [35], or ABCC10 [36]. This lack of synthetic approaches is explained by the absence of lead structures as starting point for potential synthesis and lead optimization.

Computational approaches are great tools for lead discovery and subsequent optimization with the support of organic synthesis. They have extensively been used within the past 20 years. The vast majority of reports specified structure-based retrospective computational approaches, in which observed biological effects of compounds were underpinned mostly through molecular docking experiments with cryo-EM structures or homology models. Most pronounced are, again, ABCB1 [37], [38], [39], [40], [41], [42] and ABCG2 [43], [44], [45], [46], [47], [48], [49], [50], [51], [52], [53]. Less- or under-studied ABC transporters are barely reflected in the literature. In terms of retrospective molecular docking experiments, rare exceptions are ABCB5 [54], ABCB6 [55], or ABCC10 [56], [57]. Ligand-based retrospective approaches are much less present in literature and have been described, for example, for ABCB11 [20], [21], [22], or ABCC2 [58].

**Fig. 1:**
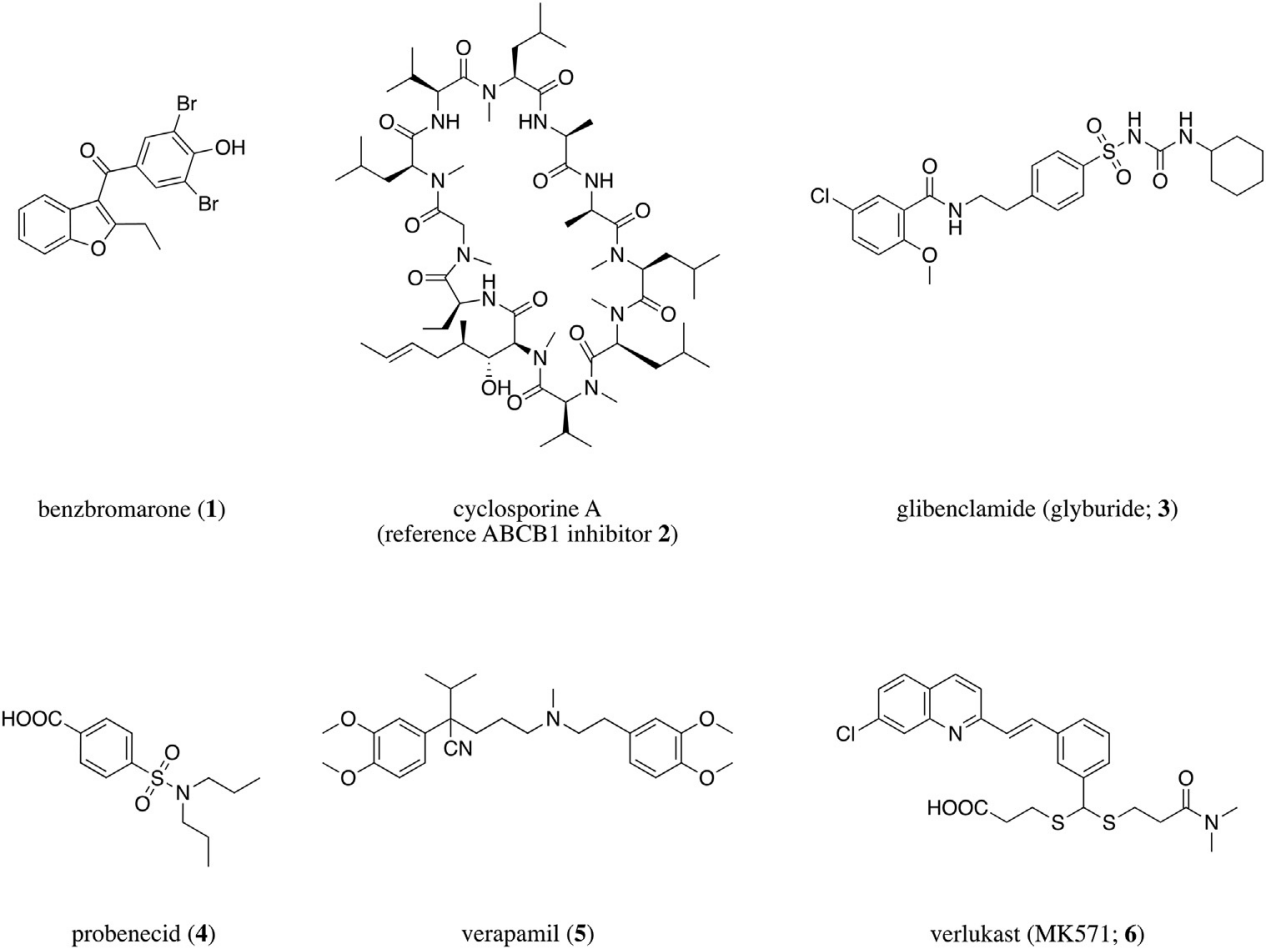
Drugs and drug-like compounds that were shown in several independent studies to be pan-ABC transporter inhibitors. Cyclosporine A (**2**) was used as standard ABCB1 inhibitor in the presented study.

Prospective approaches for the discovery of novel lead molecules are generally limited with respect to ABC transporters. Regarding structure-based design through molecular docking approaches, ABCB1 [59], [60], [61] and ABCG2 [43], [62], [63] are most pronounced, but also ABCC4 [64] or ABCC5 [65], [66] have been investigated. Prospective ligand-based design is more preferred, as it does not rely on crystal structures, cryo-EM structures, or homology models of ABC transporters. Similarity search (ABCC1 [67] or ABCC4 [64]), pharmacophore modelling (ABCB1 [61], [68], ABCB11 [69], or ABCC1 [67]), machine learning (ABCB1 [70] or ABCG2 [71]), or other pattern-based approaches [72] were demonstrated as powerful computational tools for lead identification, which eventually led often to virtual screenings and actual hit discovery [64], [67], [69]. However, these approaches always took only one transporter into account, completely leaving out the potential of multitarget inhibition. Multitargeting is a promising approach to explore under-studied ABC transporters by targeting similar or mutually overlapping binding sites [15], [73], [74]. Several pharmacological drugs have already been revealed as (weak) pan-ABC transporter inhibitors (=inhibiting several ABC transporters simultaneously), as for example, benzbromarone (**1**; ABCB1 [75], ABCB11 [20], ABCC1-6 [23], [24], [58], [76], [77], and ABCG2 [75]), cyclosporine A (**2**; ABCA1 [27], ABCB1 [16], ABCB4 [78], ABCB11 [20], ABCC1–2 [23], [58], ABCC10 [24], ABCG1–2 [75], [79]), glibenclamide (glyburide, **3**; ABCA1 [29], ABCB11 [20], ABCC1 [23], ABCC5 [80], ABCC7–9 [81], [82], [83], ABCG2 [58]), probenecid (**4**; ABCA8 [84], ABCC1–6 [23], [24], [85], [86], [87], ABCC10 [88]), verapamil (**5**; ABCA8 [84], ABCB1 [16], ABCB4–5 [54], [78], ABCB11 [89], ABCC1 [23], ABCC4 [90], ABCC10 [88], ABCG2 [58]), or verlukast (MK571, **6**; ABCA8 [84], ABCB4 [78], ABCB11 [20], ABCC1–5 [23], [58], [80], [87], [91], ABCC10–11 [24], [92], ABCG2 [58]). Fig. 1 provides the molecular formulae of the most prominent drug-like pan-ABC transporter inhibitors known until today.

**Fig. 2:**
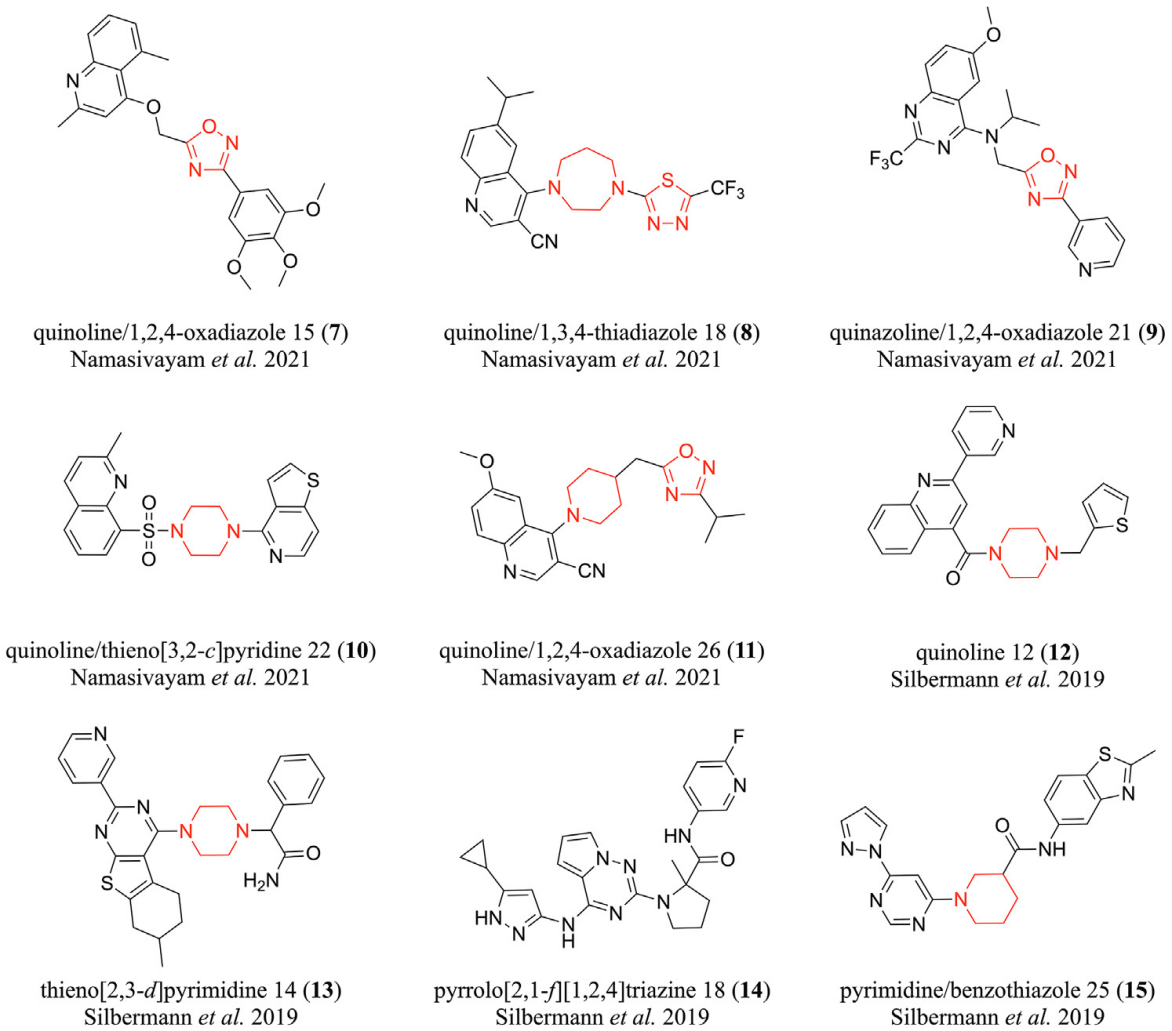
Broad-spectrum ABCB1, ABCC1, and ABCG2 inhibitors obtained from computational approaches. Compounds **7**–**11** were derived from C@PA as reported by Namasivayam *et al.* in 2021 [15]. Compounds **12**–**15** resulted from a combined ligand-based approach using similarity search and pharmacophore modelling as reported by Silbermann *et al.* in 2019 [67]. The corresponding IC_50_ values can be found in Table 1. Red mark: suggested secondary positive hits as proposed before [15]. (For interpretation of the references to colour in this figure legend, the reader is referred to the web version of this article.)

As indicated above, ABCB1, ABCC1, and ABCG2 are the most investigated and understood ABC transporters, and hence, represent model targets for the generation of pan-ABC transporter inhibitors [15]. However, even for these well-studied ABC transporters, only 133 broad-spectrum ABCB1, ABCC1, and ABCG2 inhibitors were described to date [15], [43], [45], [62], [67], [75], [93], [94], [95], [96], [97], [98], [99], [100], [101], [102], [103], [104], [105], [106], [107], [108], [109], [110], [111], [112], [113], [114], [115], [116], [117], [118], [119], [120], [121], [122], [123], [124], [125], [126], amongst which only 56 exerted their effects below 10 µM [15], [43], [45], [62], [67], [75], [93], [94], [95], [98], [99], [101], [103], [107], [109], [110], [111], [112], [113], [118], [119], [120], [121], [122], [123], [126], and only 23 showed effects at ≤5 µM [43], [62], [98], [101], [103], [109], [110], [111], [112], [119], [121], [122], [126]. There is generally a lack of highly potent ABCB1, ABCC1, and ABCG2 inhibitors and only a highly limited understanding regarding prediction and discovery of such agents. Recently, we were the first to report on a novel computer-aided pattern analysis (C@PA) approach for the prediction of potent multitarget ABCB1, ABCC1, and ABCG2 inhibitors, discovering compounds **7**–**11** (Fig. 2) [15]. As the data regarding ABCB1, ABCC1, and ABCG2 is much more advanced than toward other transporters, we continued to improve C@PA’s prediction capabilities, which is reported in the presented study.

## 2 Results and discussion

### 2.1 Basic scaffold dissection and potential positive hit identification

**Fig. 3:**
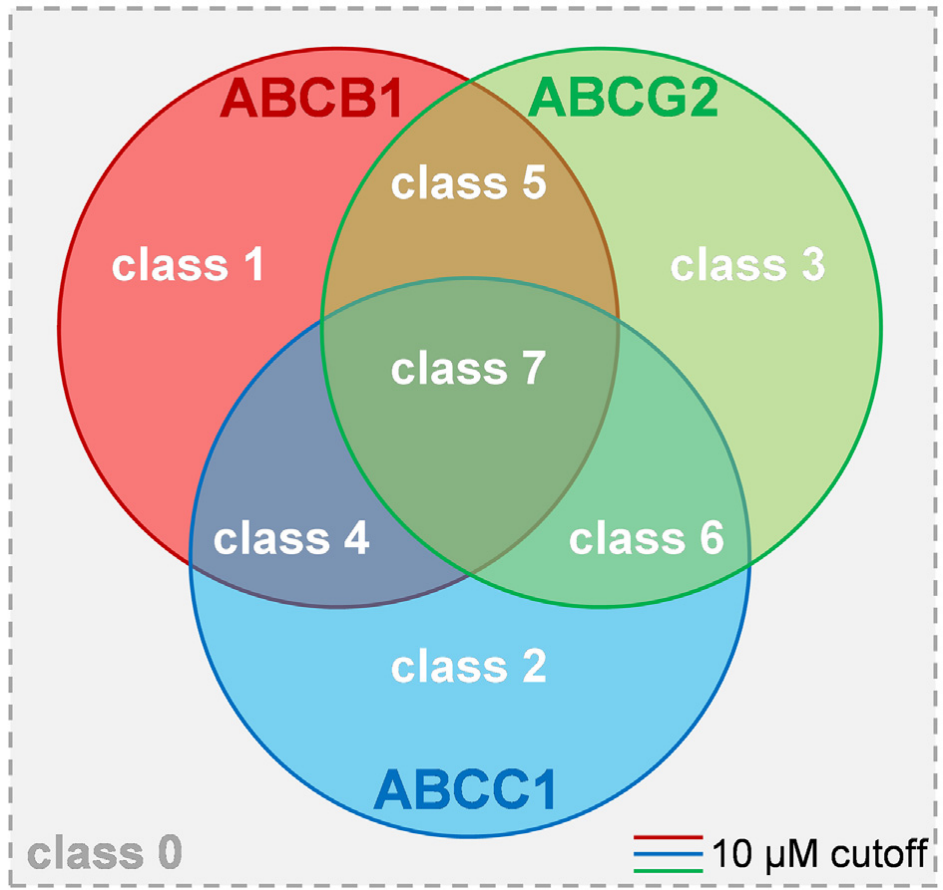
Visualization of the classification of modulators of ABCB1, ABCC1, and ABCG2 as proposed earlier [15]: ‘class 7 compounds’ are defined as triple ABCB1, ABCC1, and ABCG2 inhibitors that exert their half-maximal effect against these transporters below 10 µM. This has up to date been reported for 56 compounds [15], [43], [45], [62], [67], [75], [93], [94], [95], [98], [99], [101], [103], [107], [109], [110], [111], [112], [113], [118], [119], [120], [121], [122], [123], [126]. Amongst these molecules are the compounds revealed by C@PA, **8–9** and **11**.

In our latest report about C@PA, we identified so-called ‘multitarget fingerprints’ for the prediction of broad-spectrum ABCB1, ABCC1, and ABCG2 inhibitors amongst a manually assembled and curated initial dataset of 1,049 compounds [15]. The model was generated on the basis of (i) the identification of basic scaffolds amongst the most potent known ABCB1, ABCC1, and ABCG2 inhibitors; (ii) the definition of substructures with a positive impact regarding multitarget ABCB1, ABCC1, and ABCG2 inhibition; and (iii) the definition of substructures with a negative impact with respect to multitarget ABCB1, ABCC1, and ABCG2 inhibition. As a result, compounds **8**–**9** as well as **11** were discovered by a virtual screening as so-called ‘class 7 compounds’ (=IC_50_ values below 10 µM toward ABCB1, ABCC1, and ABCG2; Fig. 3).

In total, 5 multitarget ABCB1, ABCC1, and ABCG2 inhibitors were discovered (**7–11**) [15], which contained 5 partial structures that were suggested by us as ‘secondary positive hits’: (i) 1,2,4-oxadiazole; (ii) 1,3,4-thiadiazole; (iii) piperazine; (iv) homo-piperazine; and (v) piperidine. In the present study, we extended the positive pattern fingerprints by ‘potential positive hits’ in order to explore their impact on the inhibitory nature of molecules on ABCB1, ABCC1, and ABCG2 function in combination with already known primary positive substructures.

**Fig. 4:**
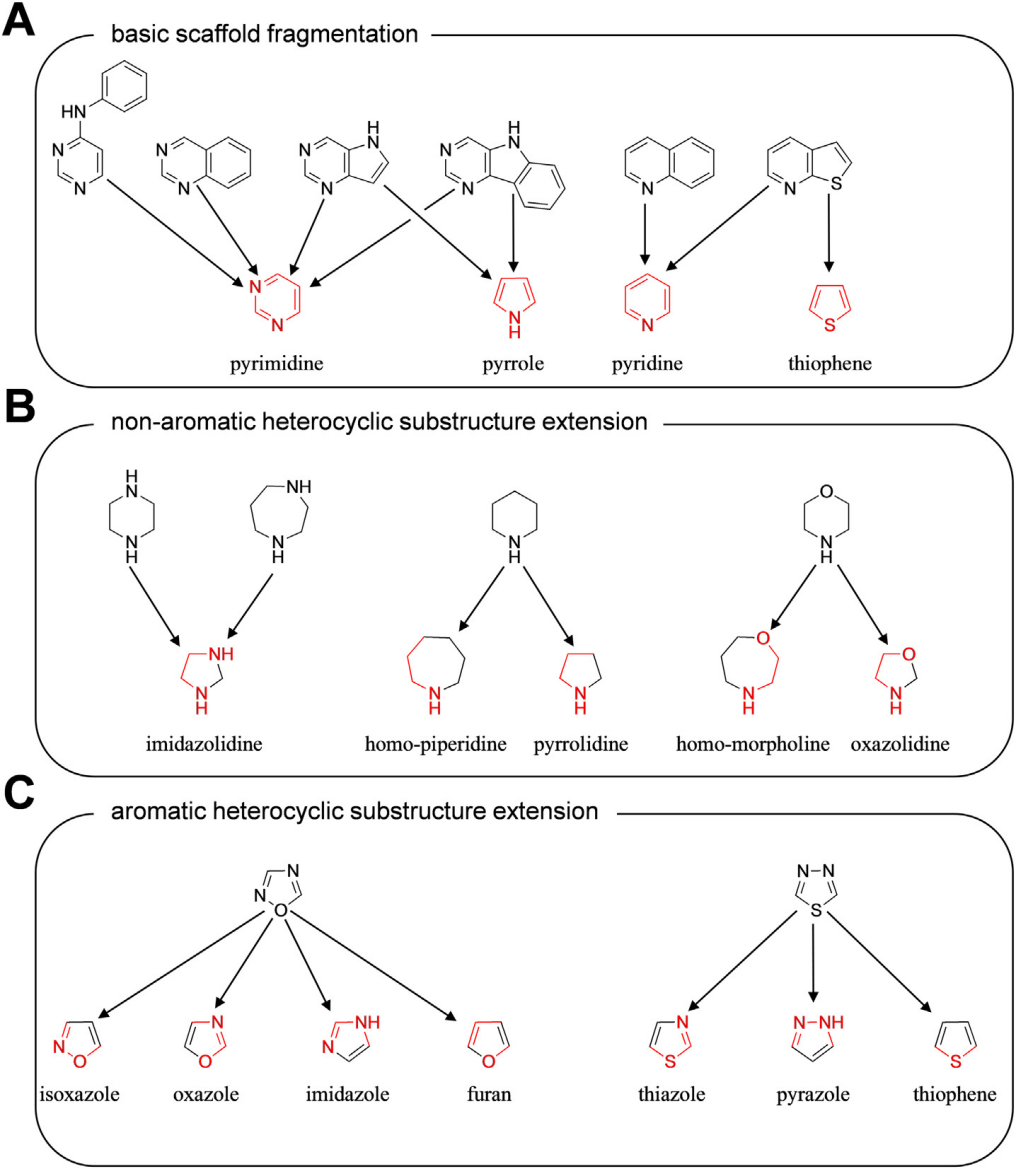
Extension of positive pattern fingerprints: (A) dissection of C@PA-derived basic scaffolds into smaller heteroaromatic units (‘Scaffold Fragmentation’); (B) deduction of non-aromatic heterocycles from C@PA-derived clear positive and secondary positive hits (‘Heterocyclic Substructure Hopping’); and (C) deduction of heteroaromatic five-membered rings from C@PA-derived secondary positive heteroaromatic substructures (‘Heteroaromatic Substructure Hopping’). Red mark: conserved part of the novel substructure. (For interpretation of the references to colour in this figure legend, the reader is referred to the web version of this article.)

As a first step, we dissected the basic scaffolds (‘Scaffold Fragmentation’; Fig. 4 A) as derived by C@PA [15], which resulted in the first extension of the positive pattern fingerprints with potential positive hits: (i) pyrimidine; (ii) pyrrole; (iii) pyridine; and (iv) thiophene. As a second step, we extended the structural variety of the non-aromatic heterocycles (‘Heterocyclic Substructure Hopping’; Fig. 4 B) as derived and proposed by C@PA: (i) imidazolidine deduced from piperazine and homo-piperazine; (ii) homo-piperidine and pyrrolidine deduced from piperidine; and (iii) homo-morpholine and oxazolidine deduced from morpholine. It must be noted that pyrrolidine was earlier identified by C@PA as a ‘clear negative hit’ [15]. However, for a detailed investigation of piperidine derivatives and their impact on ABCB1, ABCC1, and ABCG2 function, this clear negative hit needed overruling. As a final step, the two found novel aromatic substructures in compounds **7**–**11**, 1,2,4-oxadiazole and 1,3,4-thiadiazole, were extended by five-membered rings that had conserved features of the original substructure (‘Heteroaromatic Substructure Hopping’; Fig. 4 C). Hence, the following aromatic substructures were added to the potential positive hit list: (i) isoxazole; (ii) oxazole; (iii) imidazole; (iv) furan; (v) thiazole; (vi) pyrazole; and (vii) thiophene. Here again, it must be taken note that oxazole was also identified by C@PA as a clear negative hit [15], however, it was now added for a detailed evaluation of 1,2,4-oxadiazole derivatives toward their effect on ABCB1, ABCC1, and ABCG2 function. In total, the 8 'clear positive hits' as derived from C@PA [15] were extended by additional 5 suggested secondary positive hits [15] and 15 deduced potential positive hits. These 20 substructures will in the following be referred to as ‘extended positive hits’ (‘Extended Positive Pattern’).

### 2.2 Virtual screening and compound selection

The clear limit of C@PA was the prediction of ABCC1 inhibitors, as the discovered multitarget ABCB1, ABCC1, and ABCG2 inhibitors **7**–**11** were the only present ABCC1 inhibitors in the biologically evaluated set of 23 compounds [15]. To counteract this effect, we selected a virtual screening data set that favored ABCC1 inhibition as reported by us before [67]. This set of molecules comprised of 1,510 compounds that resulted from a combined virtual screening approach for the prediction of ABCC1 inhibitors. It is known that ~ 23.5% of these compounds comprised of ABCC1 inhibitors. We favored this virtual screening dataset compared to other options because the identified ABCC1 inhibitors (**12**–**15**; Fig. 2) were in parallel multitarget ABCB1, ABCC1, and ABCG2 inhibitors, which possibly increased the chance to identify novel lead molecules for broad-spectrum ABCB1, ABCC1, and ABCG2 inhibition.

**Fig. 5:**
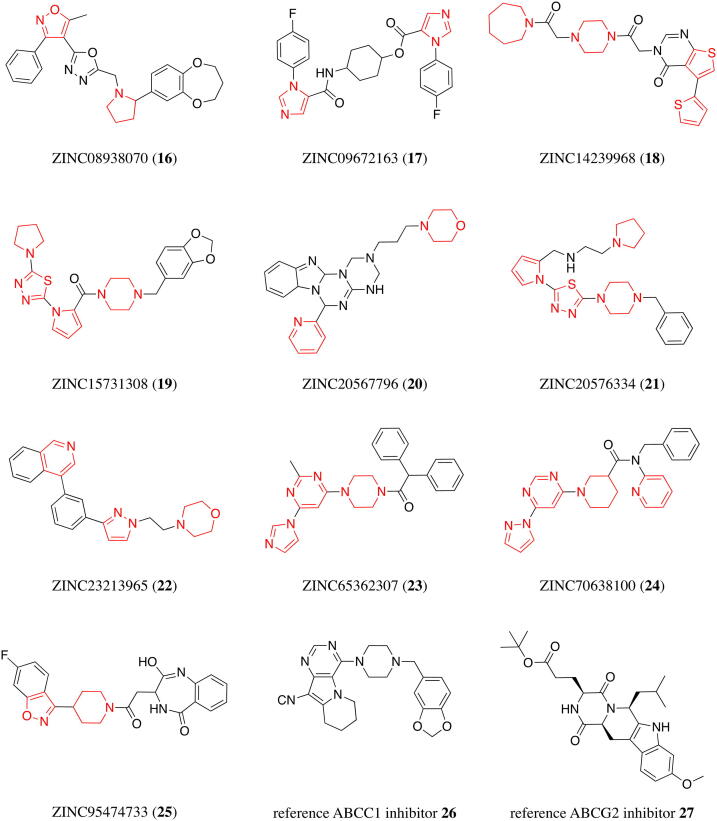
Hit molecules **16**–**25** derived from the herein presented virtual screening approach as well as the reference ABCC1 and ABCG2 inhibitors, **26** and Ko143 (**27**), respectively, used in the present study [67], [101]. The corresponding IC_50_ values of compounds **16**–**25** can be found in Table 1. Red Mark: extended positive pattern. (For interpretation of the references to colour in this figure legend, the reader is referred to the web version of this article.)

**Fig. 6:**
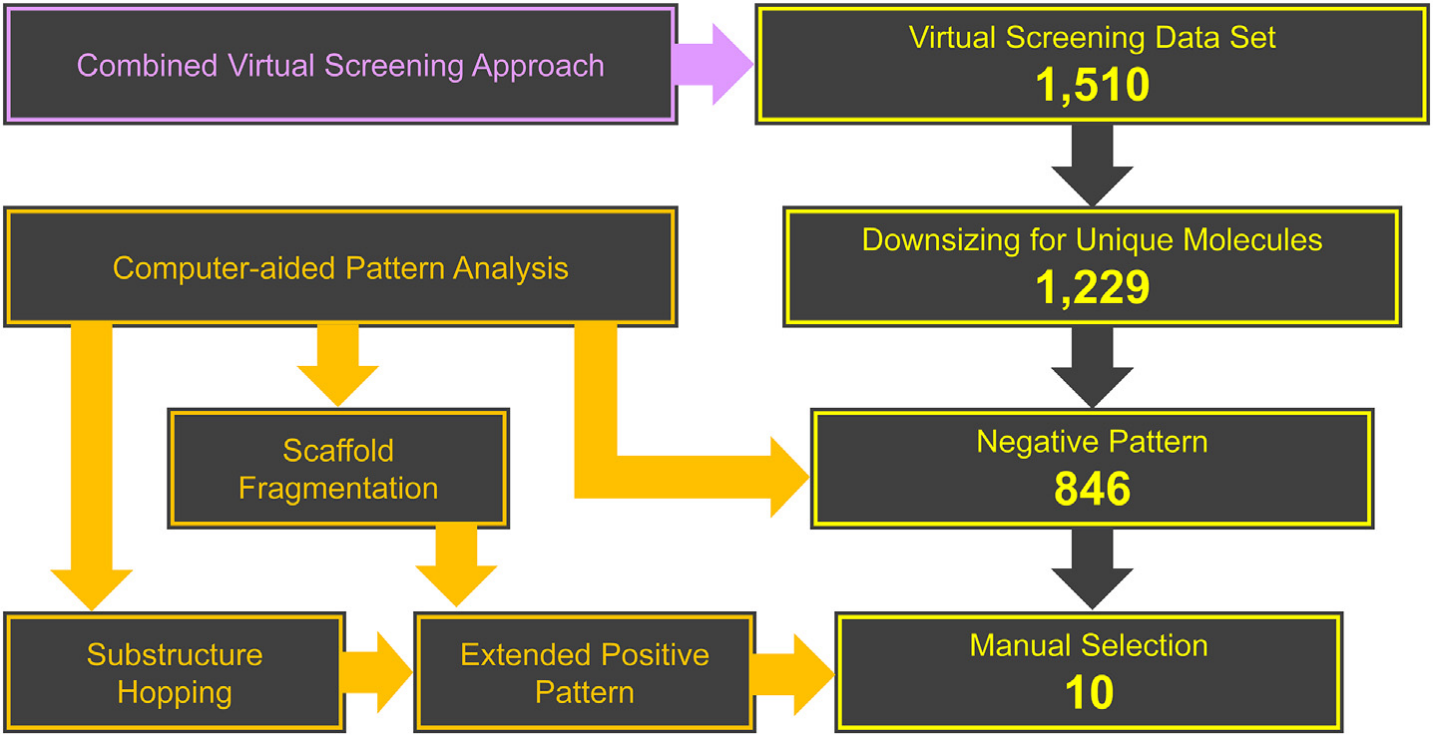
Workflow of the herein presented virtual screening approach.

As a first step, the 1,510 compounds were screened for redundant molecules in form of stereoisomers to increase the diversity of the virtual screening data set. In total, 281 were removed, resulting in 1,229 unique compounds. These were in a second step subject to the negative pattern search [15]. While 383 compounds have been eliminated, 846 remained in the virtual screening dataset. Finally, the 846 compounds were screened for the extended positive hits. At least one of these favored substructures was present in these 846 molecules with the following distribution: (i) 1 time: 29 molecules; (ii) 2 times: 277 molecules, (iii) 3 times: 356 molecules; (iv): 4 times: 149 molecules; (v) 5 times: 34 molecules; (vi): 6 times: 1 molecule. From these 846 potential broad-spectrum ABCB1, ABCC1, and ABCG2 inhibitors we manually selected and purchased 10 candidates (compounds **16**–**25**; Fig. 5) depending on manner and number of extended positive hits present and general molecular composition, as well as commercial availability and affordability at MolPort® (www.molport.com). Fig. 6 shows the virtual screening flow as exerted in this study.

### 2.3 Biological evaluation

**Fig. 7:**
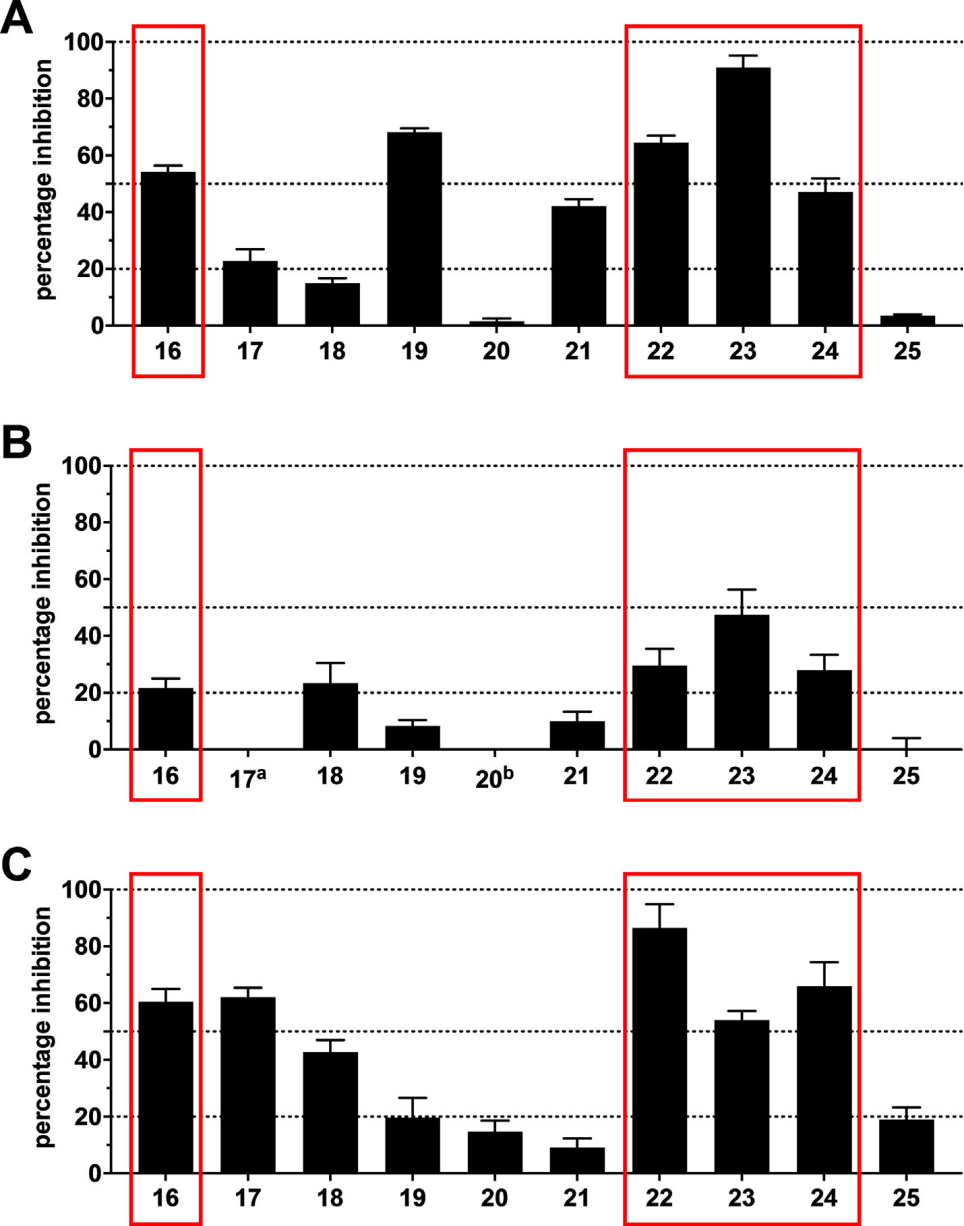
Preliminary screening of compounds **16**–**25** against ABCB1 (A), ABCC1 (B), and ABCG2 (C) in calcein AM (A and B) and pheophorbide A (C) assays, respectively, using ABCB1-overexpressing A2780/ADR (A), ABCC1-overexpressing H69AR (B), and ABCG2-overexpressing MDCK II BCRP (C) cells as described earlier [15], [43], [45], [67], [101]. The data were normalized by defining 100% inhibition by the effect value of 10 µM of the reference inhibitors **2** (ABCB1; A), **26** (ABCC1, B), and **27** (ABCG2, C) as reported earlier [67], [101]. Shown is mean ± standard error of the mean (SEM) of at least three independent experiments. Red mark: triple ABCB1, ABCC1, and ABCG2 inhibitors. ^a^ no inhibition; ^b^ apparent ABCC1 activation (effect at 10 µM: 12.7% ± 2.4%). (For interpretation of the references to colour in this figure legend, the reader is referred to the web version of this article.)

Compounds **16**–**25** were screened at 10 µM in calcein AM (ABCB1 and ABCC1) as well as pheophorbide A (ABCG2) fluorescence accumulation assays using either ABCB1-overexpressing A2780/ADR, ABCC1-overexpressing H69AR, or ABCG2-overexpressing MDCK II BCRP cells, respectively, as reported earlier [15], [43], [45], [67], [101]. Calcein AM and pheophorbide A are substrates of ABCB1 and ABCC1 as well as ABCG2, respectively, which passively diffuse into the used cells and become extruded by the corresponding ABC transporter. Inhibition of the respective transporter results in the accumulation of these substrates. Calcein AM is subsequently cleaved by intracellular esterases to the fluorescent calcein, while pheophorbide A is already fluorescent. Intracellular fluorescence was determined *via* microplate reader (calcein AM; ABCB1 and ABCC1) and flow cytometry (pheophorbide A; ABCG2), respectively. Compounds **2** (Fig. 1) and **26**–**27** (Fig. 5) were used as reference inhibitors against ABCB1, ABCC1, and ABCG2, respectively, as reported before [67], [101]. Fig. 7 provides the screening results for ABCB1 (A), ABCC1 (B), and ABCG2 (C).

As a result, 7 compounds had activities against ABCB1 (**16**–**17**, **19**, **21**–**24**), while 5 candidates inhibited ABCC1 (**16**, **18**, **22**–**24**), and 8 were active against ABCG2 (**16**–**19**, **22**–**25**). Amongst the 10 evaluated compounds, 7 multitarget ABC transporter inhibitors could be identified: 4 triple ABCB1, ABCC1, and ABCG2 inhibitors (**16**, **22**–**24**), 2 dual ABCB1 and ABCG2 inhibitors (**17**, **19**), and 1 dual ABCC1 and ABCG2 inhibitor (**18**), which represents a multitarget hit rate of 70%. This even exceeded the very high multitarget hit rate of C@PA of 60.9% as reported earlier [15]. Compounds **21** and **25** were shown to be selective ABCB1 and ABCG2 inhibitors, respectively, while compound **20** did inhibit neither of the evaluated transporters. Table 1 presents the determined IC_50_ values of the compounds that reached at least 20% [+SEM (standard error of the mean)] compared to the standard ABCB1 (**2**), ABCC1 (**26**), and ABCG2 (**27**) inhibitors.

**Table 1:** The determined IC_50_ values of compounds that resulted in an inhibition level of ≥20% [+ standard error or the mean (SEM)] in the preliminary screening (Fig. 7 A–C) determined in calcein AM (ABCB1 and ABCC1) and pheophorbide A (ABCG2) assays, respectively, applying ABCB1-overexpressing A2780/ADR, ABCC1-overexpressing H69AR, and ABCG2-overexpressing MDCK II BCRP cells, respectively, as described earlier [15], [43], [45], [67], [101]. The reference inhibitors (ABCB1: **2**; ABCC1: **26**; ABCG2: **27**) served as positive controls as already reported earlier [67], [101], defining 100% inhibition. Buffer medium served as a negative control (0%). Shown is mean ± SEM of at least three independent experiments. Light rose mark: IC_50_ values of the triple ABCB1, ABCC1, and ABCG2 inhibitors **7**–**15** as reported earlier [15], [67]; dark rose mark: within this work discovered novel multitarget ABCB1, ABCC1, and ABCG2 inhibitors.

^a^ Compound was reported before [15].

^b^ Compound was reported before [67].

^c^ Not determined due to the lack of inhibitory activity in the initial screening (Fig. 7 A–C).

**Fig. 8:**
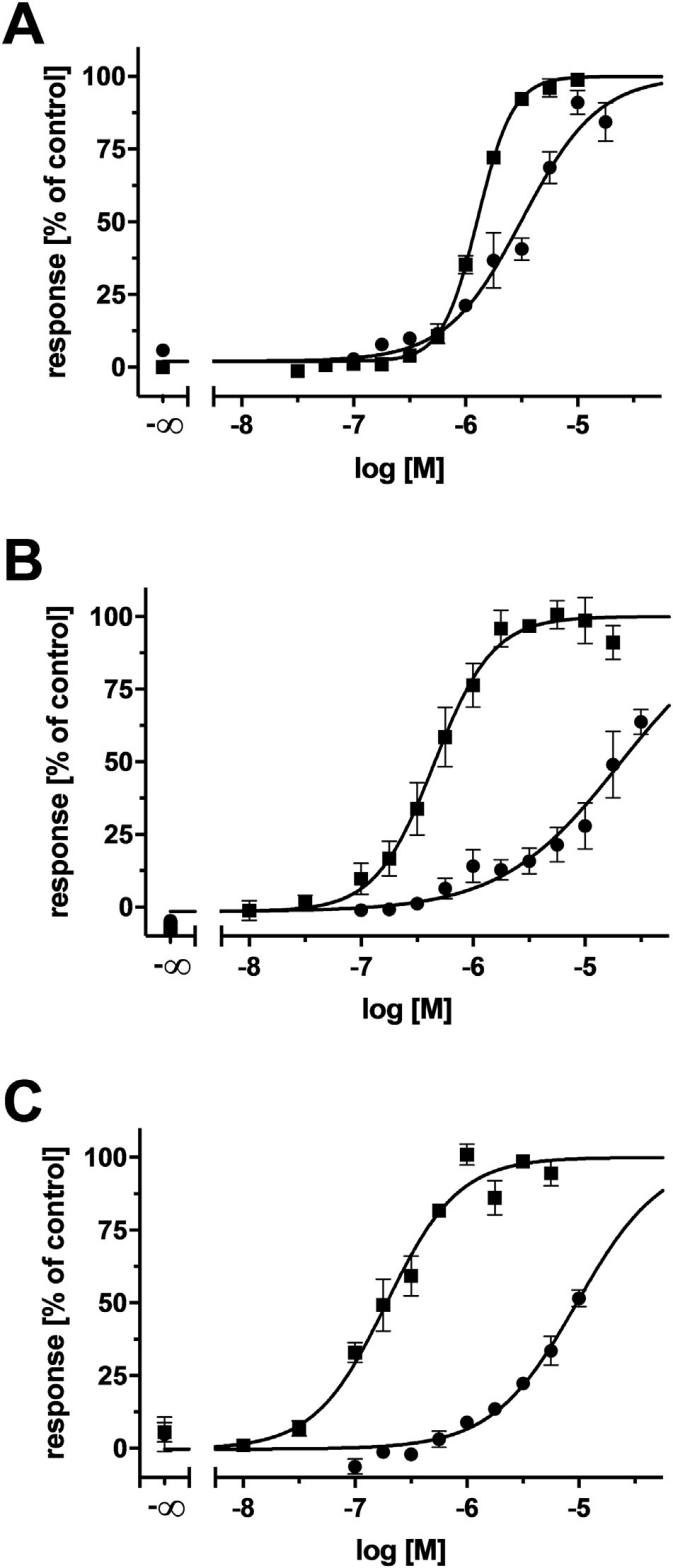
Concentration-effect curves of compound **23** (●) against ABCB1 (A), ABCC1 (B), and ABCG2 (C) as obtained in calcein AM (A and B) and pheophorbide A (C) assays, respectively, compared to the reference inhibitors **2** (A;■), **26** (B; ■), and **27** (C; ■) applying ABCB1-overexpressing A2780/ADR (A), ABCC1-overexpressing H69AR (B), and ABCG2-overexpressing MDCK II BCRP (C) cells, respectively, as reported earlier [15], [43], [45], [67], [101]. Shown is mean ± SEM of at least three independent experiments.

The discovery of 4 triple ABCB1, ABCC1, and ABCG2 inhibitors out of 10 candidates represents a biological hit rate of 40%, which was higher than the individual multitarget hit rates as reported in the combined similarity search and pharmacophore modelling approach (23.5%) and C@PA (21.7%) [15], [67]. Amongst these multitarget ABCB1, ABCC1, and ABCG2 inhibitors, compound **23** showed promising inhibitory activities against ABCB1 (4.01 µM), ABCC1 (14.8 µM), and ABCG2 (9.27 µM), almost qualifying it as a class 7 compound (Fig. 3). Besides the above mentioned 56 class 7 compounds [15], [43], [45], [62], [67], [75], [93], [94], [95], [98], [99], [101], [103], [107], [109], [110], [111], [112], [113], [118], [119], [120], [121], [122], [123], [126], further 20 compounds are known which exert their inhibitor effect against ABCB1, ABCC1, and/or ABCG2 up to 15.0 µM [15], [45], [75], [93], [98], [101], [102], [110], [113], [116], [117], [119]. Considering this, compound **23** belongs to the 76 most potent multitarget ABCB1, ABCC1, and ABCG2 inhibitors known until today. Fig. 8 depicts the concentration-effect curves of compound **23** against ABCB1 (A), ABCC1 (B), and ABCG2 (C).

### 2.4 Pharmacophore modelling

**Fig. 9:**
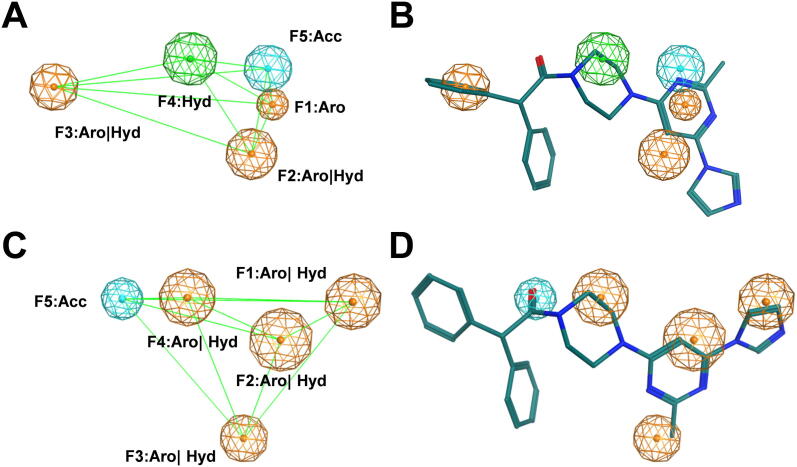
Pharmacophore model of the most potent triple ABCB1, ABCC1, and ABCG2 inhibitor presented in this work, compound **23**. The five multitarget ABCB1, ABCC1, and ABCG2 features [(i–iv) F1–F4: aromatic/hydrophobic; and (v) F5: acceptor] as reported before [15] are depicted (A), to which compound **23** was aligned to (B). In comparison, the five features for ABCC1 inhibition [(i) F1: aromatic; (ii–iii) F2 and F3: aromatic/hydrophobic; (iv) F4: hydrophobic; and (v) F5: acceptor] as reported before [67] are shown (C), and the respective conformer pose of compound **23** (D). The distances between the pharmacophore features are shown as light green lines. While nonpolar hydrogen atoms were omitted, carbon, oxygen, and nitrogen atoms were colored in green, red, and blue, respectively. (For interpretation of the references to colour in this figure legend, the reader is referred to the web version of this article.)

In our previous study, we have explored different ligand-based approaches to validate C@PA [15]. A generated pharmacophore model based on the 6 most potent and diverse class 7 compounds (Supplementary Fig. 1) showed a sensitivity value of 60.4% and a specificity value of 44.5% (C@PA: 62.5% and 90.8%, respectively). Five pharmacophore features were discovered: (i–iv) F1–F4: aromatic/hydrophobic; and (v) F5: acceptor (Fig. 9 A). In the present study, we aimed for an additional investigation of the potential binding properties of compound **23**. Hence, we performed a search on the recently presented pharmacophore model [15] for triple ABCB1, ABCC1, and ABCG2 inhibitors [15] by generating conformers of compound **23**. As can be seen in Fig. 9 B, compound **23** reflected all five pharmacophore features as derived in the multitarget pharmacophore model [15], which confirms compound **23** as a moderately potent triple ABCB1, ABCC1, and ABCG2 inhibitor. In addition, compound **23** did also reflect all five pharmacophore features as derived from the previously reported similarity search and pharmacophore modelling approach [(i) F1: aromatic; (ii–iii) F2 and F3: aromatic/hydrophobic; (iv) F4: hydrophobic; and (v) F5: acceptor; Fig. 9 C–D] [67]. This suggests that compound **23** represents a good lead molecule for further improvement *via* synthesis to gain novel potent multitarget ABCB1, ABCC1, and ABCG2 inhibitors focusing ABCC1 inhibition. Furthermore, the findings support the hypothesis of a common multitarget binding site amongst different ABC transporter subfamilies as postulated earlier [15], [74].

## 3 Conclusions

### 3.1 Statistical framework of C@PA

The aim of the present study was to extend the knowledge regarding multitarget fingerprints independent from the statistical background as reported previously [15]. This measure was necessary as it is almost impossible to change the statistical distribution of substructures amongst multitarget inhibitors (classes 4–7), specifically class 7 molecules, but also non-multitarget compounds (classes 0–3), unless a compound library of significant size (=hundreds of compounds) compared to the initial dataset of 1,049 compounds of C@PA [15] is synthesized and biologically evaluated on all three transporters. This is unlikely to happen within the next years.

The clear limit of the presented study was to discover class 7 compounds, supporting the threshold values set initially as the selection criteria of C@PA. [15]. These C@PA-derived clear positive hit and clear negative hit substructures are an important framework to obtain potent multitarget ABCB1, ABCC1, and ABCG2 inhibitors [15]. Especially the 32 clear negative hits proved to be of major importance compared to the only 8 found clear positive hits. However, the present work revealed that changes in these substructure compositions are tolerated, indicating an acceptable robustness of C@PA. This can also be visualized when comparing the initial hit rate for multitarget ABCB1, ABCC1, and ABCG2 inhibition of the virtual screening data set (23.5%) [67] with the hit rate of 40% found in the presented work, which indicates that C@PA_1.2 is an even more powerful methodology for the prediction of broad-spectrum ABCB1, ABCC1, and ABCG2 inhibitors. Strikingly, the present work demonstrated that the combination of C@PA with other computational approaches, in particular similarity search and pharmacophore modelling, led to a predictive synergism. Hence, the refinement of computer-chemical approaches with improved patterns and data sets may provide even higher biological hit rates in further developed pattern analysis models (*e.g.*, C@PA_1.X).

### 3.2 Potential of extended positive hits: under-represented substructures

Several defined extended positive hits were reflected in the discovered multitarget ABCB1, ABCC1, and ABCG2 inhibitors **16** and **22**–**24**, namely (i) pyrimidine (**24**), (ii) pyridine (**22–24**), (iii) isoxazole (**16**), (iv) imidazole (**23**), (v) pyrazole (**22** and **24**), and pyrrolidine (**16**). This discovery ultimately showed that the 8 clear positive hits as derived from C@PA [15] may indeed be supported by secondary positive hits, revealing the high potential of substructure extension in C@PA. A detailed analysis of these substructures according to their statistical distribution amongst the 133 known multitarget ABCB1, ABCC1, and ABCG2 inhibitors [15], [43], [45], [62], [67], [75], [93], [94], [95], [96], [97], [98], [99], [100], [101], [102], [103], [104], [105], [106], [107], [108], [109], [110], [111], [112], [113], [114], [115], [116], [117], [118], [119], [120], [121], [122], [123], [124], [125], [126] showed that the substructures isoxazole, imidazole, pyrazole, and pyrrolidine occurred only 1, 3, 1, and 2 times [67], [96], [106], [107], [117], [127], respectively, in these 133 compounds, and were generally only present in 1, 16, 8, and 8 molecules, respectively, of the initial dataset of 1,049 compounds as used in C@PA [15]. Our results indicate that these ‘under-represented substructures’ pose a high exploratory potential for the improvement of C@PA’s prediction capabilities and the discovery of novel pan-ABC transporter inhibitors, as their specific statistical evaluation as exerted in our previous report [15] can easily be changed with a small number of additional compounds.

### 3.3 Potential of extended positive hits: rejected putative positive substructures

The omnipresent substructures pyrimidine [15], [17] and pyridine [15] must be seen in a different light, as these cannot be regarded on their own as indicators for multitarget ABCB1, ABCC1, and ABCG2 inhibition due to their ubiquitousness. However, our results indicate that these substructures have generally a positive impact on broad-spectrum ABCB1, ABCC1, and ABCG2 inhibition, depending on the composition of and combination with other substructures. Statistically, pyrimidine and pyridine occurred 56 and 28 times, respectively, in the 133 known multitarget ABCB1, ABCC1, and ABCG2 inhibitors [15], [43], [45], [62], [67], [75], [93], [94], [95], [96], [97], [98], [99], [100], [101], [102], [103], [104], [105], [106], [107], [108], [109], [110], [111], [112], [113], [114], [115], [116], [117], [118], [119], [120], [121], [122], [123], [124], [125], [126]. In terms of class 7 compounds, 26 and 14 molecules contained pyrimidine and pyridine, respectively [15]. Indeed, pyrimidine and pyridine could not be considered as clear positive hits in our previous study [15] because many compounds of the other classes 0–6 contained these substructures as well (407 and 209 molecules, respectively). However, these ‘rejected putative positive substructures’ – which, nevertheless, resulted in class 7 molecules in a significant number – must be taken into special consideration for the further improvement of C@PA’s prediction capabilities (*e.g.*, C@PA_1.X). Besides pyrimidine and pyridine, we identified 14 more substructures from the initial data set of 1,049 compounds [15] that should be reconsidered in terms of multitarget ABCB1, ABCC1, and ABCG2 inhibition in particular, and pan-ABC transporter inhibition in general: (i) aniline; (ii) benzoyl; (iii) benzyl; (iv) cyano; (v) 9-deazapurine; (vi) ether; (vii) ethylenediamine; (viii) methoxy; (xiv) methoxyphenyl; (x) phenol; (xi) phenyl; (xii) piperazine; (xiii) pyrrole; and (xiv) resorcin. Cyano, methoxy, and piperazine were already proposed in our previous study as secondary positive hits [15]. Nevertheless, it must clearly be noted that the percentage of occurence of these particular substructures amongst class 7 compounds is rather low. However, they might support other, clearer positive indicators of broad-spectrum ABCB1, ABCC1, and ABCG2 inhibition, enhancing compound potency through their proportionate contribution and combination, which represents a high potential for further developed C@PA-derived models (*e.g.*, C@PA_1.X).

### 3.4 Outlook: the future of pan-ABC transporter modulators

The present study contributed to a major understanding of pattern analysis and possibilities to extend chemical patterns with the purpose to enhance the prediction rate to obtain biologically active compounds. The statistical distribution of certain substructures that occurred in class 7 or class 4–6 molecules in the initial data set of 1,049 compounds needs revision and re-evaluation, taking the results of the present study into account. We propose a ranking methodology to maximally increase the impact of secondary positive substructures in combination with primary positive hits for the best possible multitarget ABCB1, ABCC1, and ABCG2 inhibition. Deciphering the interconnection between manner, number, as well as the orientational composition of certain substructures and maximal possible impact on ABCB1, ABCC1, and ABCG2 will provide potential candidates for biological screening on other ABC transporters, exploring their nature, function, as well as their suitability as therapeutic or diagnostic drug targets. Furthermore, recent advances in crystallographic methodologies, such as cryo-EM, increasingly provided structural information of ABC transporters of different sub-families. This will allow for the analysis of the ‘multitarget binding site’ [15], [74] with the identified multitarget pan-ABC transporter inhibitors applying a combination of structure-based computational approaches. Using the knowledge derived from C@PA, C@PA_1.2, and potentially C@PA_1.X, new truly multitarget pan-ABC transporter modulators will be derived that could address less- and under-studied ABC transporters to tackle common and rare human diseases.

## 4 Experimental section

### 4.1 Computational analysis

#### 4.1.1 Virtual screening dataset

The virtual screening dataset of the 1,510 putative ABCC1 inhibitors was derived by a combined similarity search and pharmacophore modelling approach as described earlier [67]. In short, an initial dataset of 288 known ABCC1 inhibitors with definite IC_50_ values was collected from ChEMBL [128] and categorized [‘active’ (IC_50_ < 1 µM); ‘moderate’ (IC_50_ = 1–10 µM); ‘inactive’ (IC_50_ > 10 µM)]. Similarity search applying the FTrees algorithm [129], [130] from BioSolveIT GmbH (Sankt Augustin, Germany) was conducted with a Tanimoto coefficient (Tc) of 0.8 by which the database was analyzed according to 4 query molecules (Supplementary Fig. 2) [101], [104], [131], [132]. The flexible alignment tool as well as the MMFF94x force field implemented in MOE (version 2016.08; Chemical Computing Group ULC, Montreal, QC, Canada) were applied for pharmacophore modelling using UNICON [133] to generate the 1000 best (=quality level 3) conformers with a tolerance distance of 1.5 Å and a threshold of 50.0% conservation. Virtual screening was performed with the ZINC12 library [134] consisting of 16,403,865 molecules from which a set of 1,510 molecules as potential ABCC1 inhibitors resulted.

#### 4.1.2 Computer-aided pattern analysis (C@PA)

The computer-aided pattern analysis (C@PA) to predict multitarget ABCB1, ABCC1, and ABCG2 inhibitors was very recently reported [15]. In short, a manually assembled initial dataset of 1,049 compounds that have at least once been assessed for their inhibitory power against ABCB1, ABCC1, and ABCG2 was categorized [‘active’ (IC_50_ < 10 µM); ‘inactive’ (IC_50_ ≥ 10 µM)] and classified as class0: inactive compounds; class 1: selective ABCB1 inhibitors; class 2: selective ABCC1 inhibitors; class 3: selective ABCG2 inhibitors; class 4: dual ABCB1 and ABCG2 inhibitors; class 5: dual ABCB1 and ABCG2 inhibitors; class 6: dual ABCC1 and ABCG2 inhibitors; and class 7: triple ABCB1, ABCC1, and ABCG2 inhibitors; Fig. 3). In total, 48 class 7 compounds were identified and analyzed for their basic scaffolds [(i) 4-anilinopyrimidine; (ii) quinazoline; (iii) pyrrolo[3,2-*d*]pyrimidine; (iv) pyrimido[5,4-*b*]indole; (v) quinoline; and (vi) thieno[2,3-*b*]pyrimidine] using the Structure-Activity-Report (SAReport) tool [135] implemented in MOE (version 2019.01). InstantJChem (version 20.15.9) was applied to statistically analyze the initial dataset of 1,049 compounds for 308 commonly occurring chemical substructures [136] and their distribution amongst classes 0–7. ‘Clear positive hits’ [‘Positive Pattern’; (i) isopropyl; (ii) amino; (iii) carboxylic acid ethyl ester; (iv) indole; (v) 3,4,5-trimethoxyphenyl; (vi) morpholine; (vii) thieno[2,3-*b*]pyrimidine; (viii) sulfone] and ‘clear negative hits’ [‘Negative Pattern’; (i) *tert*-butyl; (ii) vinyl; (iii) cyclopropyl; (iv) cyclohexyl; (v) anellated cyclopropyl; (vi) anellated cycloheptyl; (vii) dimethylamino; (viii) diethylamino; (ix) nitro; (x) pyrrolidine; (xi) methylene hydroxy; (xii) ethylene hydroxy; (xiii) oxolane; (xiv) carboxylic acid; (xv) carboxylic acid methyl ester; (xvi) biphenyl; (xvii) stilbene; (xviii) 1,2,3-triazole; (xix) 1,2,4-triazole; (xx) tetrazole; (xxi) pyrido[2,3-*d*]pyrimidine; (xxii) 1,3-dihydroisobenzofuran; (xxiii) chalcone; (xxiv) hydroquinone; (xxv) 2-methoxyphenyl; (xxvi) 3-methoxyphenyl; (xxvii) 2,5-dimethoxyphenyl; (xxviii) 3,5-dimethoxyphenyl; (xxix) unsubstituted thioamide; (xxx) oxazole; (xxxi); urea; (xxxii) thiourea)] were identified. Virtual screening was performed with the ENAMINE REAL drug-like® compound library consisting of 15,547,091 molecules and a set of 1,505 molecule as potential broad-spectrum ABCB1, ABCC1, and ABCG2 inhibitors resulted, from which compounds **7**–**11** were discovered. Five partial structures were identified amongst these compounds which could be suggested as ‘secondary positive hits’: (i) 1,2,4-oxadiazole; (ii) 1,3,4-thiadiazole; (iii) piperazine; (iv) homo-piperazine; and (v) piperidine.

#### 4.1.3 Scaffold fragmentation, substructure hopping, virtual screening, and compound selection

The C@PA-derived basic scaffolds were dissected using ChemDraw Pro [version 17.1.0.105 (19)] to (i) pyrimidine, (ii) pyrrole, (iii) pyridine, and (iv) thiophene and added to the extended positive hit list. Moreover, the non-aromatic heterocycles piperazine, piperidine, and morpholine were extended to (i) imidazolidine, (ii) homo-piperidine, (iii) pyrrolidine, (iv) homo-morpholine, and (v) oxazolidine. The aromatic substructures 1,2,4-oxadiazole and 1,3,4-thiadiazole were extended to (i) isoxazole, (ii) oxazole, (iii) imidazole, (iv) furan, (v) thiazole, (vi) pyrazole, and (vii) thiophene, and added to the extended positive hit list. In total, 29 extended positive hit substructures including the 8 clear positive hits as defined by C@PA [15] resulted. Subsequently, the 1,510 molecules derived from the combined similarity search and pharmacophore modelling approach [67] were subject to a clear negative hit exclusion (except for pyrrolidine and oxazole), with eventual extended positive hit screening. Depending on price and availability, the 10 candidates **16**–**25** were manually selected from the residual dataset of 846 potential multitarget ABCB1, ABCC1, and ABCG2 inhibitors and purchased at MolPort® (http://www.molport.com): compound **16** (Name: 2-((2-(3,4-dihydro-2H-benzo[b][1,4]dioxepin-7-yl)pyrrolidin-1-yl)methyl)-5-(5-methyl-3-phenyl-isoxazol-4-yl)-1,3,4-oxadiazole; ZINC ID: 08938070; MolPort® ID: 005–547-575; Link: https://www.molport.com/shop/moleculelink/XFQPDVQEQZTEIY-UHFFFAOYSAN/5547575; Supplier: ENAMINE Ltd.®; Cataloge No.: Z103927872; SMILES: CC1=C(C2=NN=C(O2)CN3CCCC3C4=CC5=C(OCCCO5)C=C4)C(C6=CC=CC=C6)=NO1; purity: ≥90%; compound **17** (Name: 4-(1-(4-fluorophenyl)-1H-imidazole-5-carboxamido)cyclohexyl 1-(4-fluorophenyl)-1H-imidazole-5-carboxylate; ZINC ID: 09672163; MolPort® ID: 004–504-763 Link: https://www.molport.com/shop/moleculelink/PAIVADUCKBGHFQ-UHFFFAOYSA-N/4504763; Supplier: Ukr-OrgSynthesis Ltd.®; Cataloge No.: PB169991190; SMILES: FC1=CC=C(N2C=NC=C2C(NC3CCC(OC(C4=CN=CN4C5=CC=C(F)C=C5)=O)CC3)=O)C=C1; purity: ≥90%; compound **18** (Name: 3-(2-(4-(2-(azepan-1-yl)-2-oxoethyl)piperazin-1-yl)-2-oxoethyl)-5-(thiophen-2-yl)thieno[2,3-*d*]pyrimidin-4(3H)-one; ZINC ID: 14239968; MolPort® ID: 005–770-351; Link: https://www.molport.com/shop/moleculelink/LCTNJTHHFHNEAA-UHFFFAOYSA-N/5770351; Supplier: UkrOrgSynthesis Ltd.®; Cataloge No.: PB146323516; SMILES: O=C(N1CCN(CC1)CC(N2CCCCCC2)=O)CN3C=NC4=C(C3=O)C(C5=CC=CS5)=CS4; purity: ≥90%; compound **19** (Name: (4-(benzo[d][1,3]dioxol-5-ylmethyl)piperazin-1-yl)(1-(5-(pyrrolidin-1-yl)-1,3,4-thiadiazol-2-yl)-1H-pyrrol-2-yl)methanone; ZINC ID: 15731308; MolPort® ID: 007–821-780; Link: https://www.molport.com/shop/moleculelink/YMCNSQXIHXWXTB-UHFFFAOYSA-N/7821780; Supplier: ChemDiv Inc.®; Cataloge No.: G015-0322; SMILES: O=C(C1=CC=CN1C2=NN=C(N3CCCC3)S2)N4CCN(CC4)CC5=CC=C6OCOC6=C5; purity: ≥90%; compound **20** (Name: 4-(3-(6-(pyridin-2-yl)-4,6,7a,12a-tetrahydro-1H-benzo[4,5]imidazo[1,2-*a*][1,3,5]triazino[1,2-*c*][1,3,5]triazin-2(3H)-yl)propyl)morpholine; ZINC ID: 20567796; MolPort® ID 005–912-631; Link: https://www.molport.com/shop/moleculelink/FVOLKPQFOHSUDIUHFFFAOYSAN/5912631; Supplier: Vitas-M Laboratory Ltd.®; Cataloge No.: STK790135; SMILES: N1(CCOCC1)CCCN2CNC3=NC(N4C(N3C2)N=C5C=CC=CC45)C6=CC=CC=N6; purity: ≥90%; compound **21** (Name: N-((1-(5-(4-benzylpiperazin-1-yl)-1,3,4-thiadiazol-2-yl)-1H-pyrrol-2-yl)methyl)-2-(pyrrolidin-1-yl)ethan-1-amine; ZINC ID: 20576334; MolPort® ID: 007–776-896; Link: https://www.molport.com/shop/moleculelink/AMGDZJNOUFPCEC-UHFFFAOYSA-N/7776896; Supplier: ChemDiv Inc.®; Cataloge No.: E985-0683; SMILES: N1(CCCC1)CCNCC2=CC=CN2C3=NN=C(N4CCN(CC4)CC5=CC=CC=C5)S3; purity: ≥90%; compound **22** (Name: 4-(2-(3-(3-(isoquinolin-4-yl)phenyl)-1H-pyrazol-1-yl)ethyl) morpholine; ZINC ID: 23213965; MolPort® ID: 005–039-609; Link: https://www.molport.com/shop/moleculelink/4–3-1–2-morpholin-4-yl-ethyl-1H-pyrazol-3-yl-phenyl-isoquinoline/5039609; Supplier: ChemBridge Corporation®; Cataloge No.: 25121718; SMILES: N1(C=CC(C2=CC=CC(C3=CN=CC4=CC=CC=C34)=C2)=N1)CCN5CCOCC5; purity: ≥90%; compound **23** (Name: 1-(4-(6-(1H-imidazol-1-yl)-2-methylpyrimidin-4-yl)piperazin-1-yl)-2,2-diphenylethan-1-one; ZINC ID: 65362307; MolPort® ID: 016–587-938; Link: https://www.molport.com/shop/moleculelink/DFWDBEHMZWCBIJ-UHFFFAOYSA-N/16587938; Supplier: ChemBridge Corporation®; Cataloge No.: 9210464; SMILES: CC1=NC(N2C=CN=C2)=CC(N3CCN(C(C(C4=CC=CC=C4)C5=CC=CC=C5)=O)CC3)=N1; Purity: ≥90%; compound **24** (Name: 1-(6-(1H-pyrazol-1-yl)pyrimidin-4-yl)-N-benzyl-N-(pyridin-2-yl)piperidine-3-carboxamide; ZINC ID: 70638100; MolPort® ID: 019–920-623; Link: https://www.molport.com/shop/moleculelink/HDSFZCOIEQTPRF-UHFFFAOYSA-N/19920623; Supplier: Life Chemicals Inc.®; Catalog No.: F6175-0779; SMILES: O=C(N(C1=CC=CC=N1)CC2=CC=CC=C2)C3CCCN(C4=CC(N5C=CC=N5)=NC=N4)C3; purity: ≥90%; compound **25** (Name: 3-(2-(4-(6-fluorobenzo[d]isoxazol-3-yl)piperidin-1-yl)-2-oxoethyl)-2-hydroxy-3,4-dihydro-5H-benzo[e][1,4]diazepin-5-one; ZINC ID: 95474733; MolPort® ID: 027–849-694; Link: https://www.molport.com/shop/moleculelink/SBJDVQXJQQGZMH-UHFFFAOYSA-N/27849694; Supplier: Vitas-M Laboratory Ltd.®; Cataloge No.: STL312371; SMILES: OC1=NC2=C(C(NC1CC(N3CCC(C4=NOC5=C4C=CC(F)=C5)CC3)=O)=O)C=CC=C2; purity: ≥90%.

### 4.2 Biological evaluation

#### 4.2.1 Chemicals

The reference ABCB1 inhibitor **2** as well as the reference ABCG2 inhibitor **27** were purchased from Tocris Bioscience (Bristol, UK). The standard ABCC1 inhibitor **26** was synthesized as described earlier [101]. Calcein AM and pheophorbide A were obtained from Calbiochem [EMD Chemicals (San Diego, USA), supplied by Merck KgaA (Darmstadt, Germany)]. All other chemicals were purchased from Carl Roth (Karlsruhe, Germany), Merck KgaA (Darmstadt, Germany), or Sigma-Aldrich (Taufkirchen, Germany). Compounds **16**–**26** were stored as 10 mM stock solutions at −20 °C, and dilution series as well as the in-experiment cell culture was performed with Krebs-HEPES buffer [KHB; 118.6 mM NaCl, 4.7 mM KCl, 1.2 mM KH_2_PO_4_, 4.2 mM NaHCO_3_, 1.3 mM CaCl_2_, 1.2 mM MgSO_4_, 11.7 mM D-glucose monohydrate, and 10.0 mM HEPES (2-[4-(2-hydroxyethyl)piperazin-1-yl]ethanesulfonic acid) in doubly distilled water, which was finally adjusted to pH 7.4 with NaOH and sterilized with 0.2 µm membrane filters].

#### 4.2.2 Cell culture

The ABCB1-overexpressing A2780/ADR cells were delivered by European Collection of Animal Cell Culture (ECACC, no. 93112520) and cultivated using RPMI-1640 medium (PAN-Biotech GmbH, Aidenbach, Germany) complemented with fetal bovine serum (FCS; 10%; PAN-Biotech GmbH, Aidenbach, Germany), streptomycin (50 µg/µL; PAN-Biotech GmbH, Aidenbach, Germany), penicillin G (50 U/mL; PAN-Biotech GmbH, Aidenbach, Germany), as well as L-glutamine (2 mM; PAN-Biotech GmbH, Aidenbach, Germany). The ABCC1-overexpressing H69AR cells were provided by American Type Culture Collection (ATCC CRL-11351), which were cultured with RPMI-1640 medium to which FCS (20%), streptomycin (50 µg/µL), penicillin G (50 U/mL), as well as L-glutamine (2 mM) were added. Dr. A. Schinkel (The Netherlands Cancer Institute, Amsterdam, The Netherlands) generously provided the ABCG2-overexpressing MDCK II BCRP cells, which were cultivated in Dulbecco's modified eagle medium (DMEM; Sigma Life Science, Steinheim, Germany) complemented with FCS (10%), streptomycin (50 µg/µL), penicillin G (50 U/mL), as well as L-glutamine (2 mM). The cell lines were stored in a mixture of 90% cell culture medium and 10% DMSO under liquid nitrogen, while cultivation was performed at 37 °C under 5% CO_2_-humidified atmosphere. After a confluence of 90% was reached, the cells were harvested using a 0.05%/0.02% trypsin-EDTA solution (PAN-Biotech GmbH, Aidenbach, Germany). The processing included centrifugation in a 50 mL falcon (Greiner Bio-One, Frickenhausen, Germany) at 266×*g* and 4 °C for 4 min (Avanti J-25, Beckmann Coulter, Krefeld, Germany), removal of the supernatant, resuspension in fresh cell culture media, cell counting (CASY TT cell counter with 150 µm capillary, Schärfe System GmbH, Reutlingen, Germany), as well as seeding of cells for either sub-culturing or biological testing.

#### 4.2.3 Calcein AM assay

The inhibitory activity against ABCB1 and ABCC1 was evaluated in a calcein AM assay as reported earlier [15], [67], [101]. Compounds **16**–**25** were added into a 96-well flat-bottom clear plate (Greiner, Frickenhausen, Germany) at a concentration of 100 µM and 160 µL of cell suspension containing either ABCB1-overexpressing A2780/ADR (30,000 cells/well) or ABCC1-overexpressing H69AR (60,000 cells/well) cells were added. The incubation period at 37 °C under 5% CO_2_-humidified atmosphere lasted 30 min before 20 µL of a 3.125 µM calcein AM was added to each well, subsequently followed by measurement of fluorescence increase at an excitation wavelength of 485 nm and an emission wave length of 520 nm in 60 sec intervals for 1 h in either POLARstar and FLUOstar Optima microplate readers (BMG Labtech, software versions 2.00R2/2.20 and 4.11-0; Offenburg, Germany). The slope values from the linear fluorescence increase revealed the effect value which has been normalized to the effect value of 10 µM of either compounds **2** (ABCB1) or **26** (ABCC1). As candidates **16**–**17**, **19**, and **21**–**24** as well as **16**, **18**, **22**–**24** resulted in significant inhibition (20% + SEM) of ABCB1 and ABCC1, respectively, full-blown concentration-effect curves were generated and IC_50_ values were calculated applying GraphPad Prism (version 8.4.0, San Diego, CA, USA) using the statistically preferred model (three- or four-parameter logistic equation).

#### 4.2.4 Pheophorbide A assay

The inhibitory activity against ABCG2 was evaluated in a pheophorbide A assay as reported earlier [15], [67]. Each well of a flat-bottom clear 96 well plate was complemented with 20 µL of either of the compounds **16**–**25** (100 µM), 160 µL of ABCG2-overexpressing MDCK II BCRP cells (45,000 cells/well), as well as 20 µL of a pheophorbide A solution (5 µM), subsequently incubating the plate at 37 °C in a 5% CO_2_-humidified atmosphere for 120 min. The effect values of compounds **16**–**25** were measured *via* flow cytometry [Guava easyCyte^TM^ HT, (Merck Millipore, Billerica, MA, USA; excitation: 488 nm; emission: 695/50 nm)] and compared to the effect value of 10 µM of compound **27**. As compounds **16**–**19** and **22**–**25** resulted in significant inhibition against ABCG2 (20% + SEM), complete concentration-effect curves were generated as described in 4.2.2.

### 4.3 Retrospective pharmacophore analysis

In our earlier study, a pharmacophore model was generated to evaluate the performance of C@PA [15]. This model generation has been accomplished on the basis of the 6 most potent and diverse ABCB1, ABCC1, and ABCG2 inhibitors (Supplementary Fig. 1) [43], [99], [101], [103], [110] by aligning these molecules using the flexible alignment tool as described in 4.1.1 applying MOE (version 2019.01) [15]. The best alignment was selected and the pharmacophore model was generated using the consensus method implemented in the Pharmacophore Query Editor with a threshold value of 50.0% and a tolerance distance of 1.2 Å. The conformers of the most potent multitarget ABCB1, ABCC1, and ABCG2 inhibitor in this work, compound **23**, were generated using the conformational search tool by selecting the stochastic search method implemented in MOE 2019.01. The default parameters were applied for the conformational search with a maximum limit of 10,000.

## CRediT authorship contribution statement

**Vigneshwaran Namasivayam:** Conceptualization, Methodology, Software, Validation, Formal analysis, Investigation, Data curation, Writing - original draft, Writing - review & editing, Visualization, Supervision. **Katja Silbermann:** Investigation, Writing - review & editing. **Jens Pahnke:** Resources, Writing - review & editing, Funding acquisition. **Michael Wiese:** Resources, Supervision, Writing - review & editing. **Sven Marcel Stefan:** Conceptualization, Methodology, Investigation, Data curation, Writing - original draft, Writing - review & editing, Visualization, Supervision, Project administration, Funding acquisition.

## Declaration of Competing Interest

The authors declare that they have no known competing financial interests or personal relationships that could have appeared to influence the work reported in this paper.
