# Supplementary material for: Scaffold fragmentation and substructure hopping reveal potential, robustness, and limits of computer-aided pattern analysis (C@PA)
Source: Comput Struct Biotechnol J. 2021 May 10;19:3269–83. doi: 10.1016/j.csbj.2021.05.018 (PMC8193046; doi:10.1016/j.csbj.2021.05.018)
Supplement: Supplementary data 1 [file mmc1.docx]

**Scaffold fragmentation and substructure hopping reveal potential, robustness, and limits of computer-aided pattern analysis (C@PA)**

Vigneshwaran Namasivayam^a^, Katja Silbermann^a^, Jens Pahnke^b,c,d,e^, Michael Wiese^a^, Sven Marcel Stefan*^,a,b,f^

^a^ Department of Pharmaceutical and Cellbiological Chemistry, Pharmaceutical Institute, University of Bonn, An der Immenburg 4, 53121 Bonn, Germany

^b^ Department of Neuro-/Pathology, University of Oslo and Oslo University Hospital, Sognsvannsveien 20, 0372 Oslo, Norway

^c^ LIED, University of Lübeck, Ratzenburger Allee 160, 23538 Lübeck, Germany

^d^ Department of Pharmacology, Faculty of Medicine, University of Latvia, Jelgavas iela 1, 1004 Rīga, Latvia

^e^ Department of Bioorganic Chemistry, Leibniz-Institute of Plant Biochemistry, Weinberg 3, 06120 Halle, Germany

^f^ Cancer Drug Resistance and Stem Cell Program, University of Sydney, Kolling Builging, 10 Westbourne Street, Sydney, New South Wales 2065, Australia.

* Corresponding Author: Sven Marcel Stefan (s.m.stefan@medisin.uio.no)

Phone: +47 230 71468

**Supplementary Material**

**Supplementary Figure 1** Six query molecules [43, 99, 101, 103, 110] used for generation of the multitarget ABCB1, ABCC1, and ABCG2 pharmacophore modelling as reported earlier [15]

**Supplementary Figure 2** Four query molecules [101, 104, 131, 132] used in the previously described combined virtual screening approach applying similarity search and pharmacophore modelling [67].
